# Quantitative analysis of metal artifact reduction using the auto-edge counting method in cone-beam computed tomography

**DOI:** 10.1038/s41598-020-65644-3

**Published:** 2020-06-01

**Authors:** Young Hyun Kim, Chena Lee, Sang-Sun Han, Kug Jin Jeon, Yoon Joo Choi, Ari Lee

**Affiliations:** Department of Oral and Maxillofacial Radiology, Yonsei University College of Dentistry, Seoul, South Korea

**Keywords:** Radiography, Three-dimensional imaging

## Abstract

The metal artifact reduction (MAR) algorithm is used in most CBCT unit to reduce artifact from various dental materials. The performance of MAR program of a CBCT unit according to the dental material type under different imaging mode was evaluated as introducing automatic quantification of the amount of artifact reduced. Four customized phantoms with different dental prostheses (amalgam, gold, porcelain-fused-metal, zirconia) underwent CBCT scanning with and without the MAR option. The imaging was performed under varied scanning conditions; 0.2 and 0.3 mm^3^ voxel sizes; 70 and 100 kVp. The amount of artifacts reduced by each prosthesis and scanning mode automatically counted using canny edge detection in MATLAB, and statistical analysis was performed. The overall artifact reduction ratio was ranged from 17.3% to 55.4%. The artifact caused by the gold crown was most effectively reduced compared to the other prostheses (p < 0.05, Welch’s ANOVA analysis). MAR showed higher performance in smaller voxel size mode for all prostheses (p < 0.05, independent t-test). Automatic quantification efficiently evaluated MAR performance in CBCT image. The impact of MAR was different according to the prostheses type and imaging mode, suggesting that thoughtful consideration is required when selecting the imaging mode of CBCT.

## Introduction

Since cone-beam computed tomography (CBCT) entered clinical use in the early 1990s^1^, it has been considered a powerful tool in dental diagnosis and treatment planning. Although CBCT can provide three-dimensional images similar to those obtained using conventional computed tomography (CT), it is more susceptible to artifacts due to its low radiation dose and cone-shaped X-ray source^2,3^.

In dentistry, various type of metallic materials such as dental implants, gold, and porcelain-fused-metal (PFM) have been used for restorations. These dental prostheses generally consist of materials with a high atomic number. The imaging system such as CT or CBCT uses x-ray beam with polychromatic energies and selective attenuation of lower energy photons occurs when the beam passes dental prostheses^4,5^. This phenomenon causes several types of image artifact and one of them, the streaking artifact, appears as dark and bright band across the image and degrades the image quality to the point of being diagnostically unusable.

Recently, CBCT devices with metal artifact reduction (MAR) applications have been developed^6–8^. The MAR algorithm is applied in the process of image reconstruction and reduces image distortion such as darkened areas caused by losses of gray values and bright streak artifacts^5,9^. The amount of artifact varies depending on the type, shape and size of dental materials. It is also known to be affected by the scanning conditions such as tube voltage, tube current, field of view (FOV) and voxel size^10–13^. Yet, the correlation between those factors and MAR algorithms has not been studied much. Further evaluation of the exposure condition and the MAR effect should be performed. Given that clinicians can adjust the exposure parameters in dental CBCT system according to the patient condition and diagnostic purposes, the information on optimal exposure conditions associated with MAR applied system should be elucidated in order to get the most benefit from the algorithm^14,15^.

Previous researchers used a method based on gray-level measurements in the region of interest (ROI) of CBCT images to quantitatively evaluate the efficacy of MAR algorithms^16–19^. However, the usage of gray values for quantitative measurements may not be recommended for CBCT images, since the accuracy of the gray scale in CBCT is not constant and is sensitive to image artifacts and scatter noise^3,19,20^. In CBCT which obtains the whole object volume at once, scattering x-rays are mostly reflected as noise in the individual image section. In contrary, CT obtains images slice by slice and most scattering rays escape not to have much influence on the image slice. Thus, gray value measurements are not constant between different CBCT machines, unlike CT^20,21^.

To overcome these shortcomings, we investigated metal-induced streaking artifacts that affected entire CBCT data sets by using the modified Canny edge detection algorithm to count the number of streak artifacts. Canny edge detection is known to be a precise algorithm for defining the outline features of an object in an image^22^. More specifically, we used fabricated mandibular arch-shape phantoms with four dental prostheses, which replicated patients’ oral conditions.

Thus, we aimed to investigate the performance of the MAR program of a CBCT unit with various dental prostheses under different exposure conditions and voxel resolutions and to introduce the edge detection method to determine whether the metal artifact would be reduced.

## Materials and Methods

### Dental arch phantom fabrication

**Figure 1:**
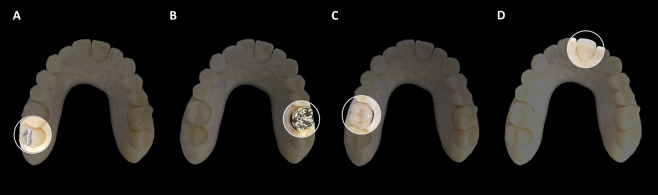
The arch-shape phantoms with four different types of prostheses. (**A**) Dental amalgam restoration on the right second molar, (**B**) a gold crown on the left first molar, (**C**) a porcelain-fused metal crown consisting of nickel-chromium alloy on the right first molar, and (**D**) a zirconia crown on the left central incisor.

Four arch-shape phantoms similar to patients’ oral conditions were fabricated using resin material (RAYDEMT C&B, Ray Co., Ltd., Gyeonggi-Do, Korea) with a 3D printer (RAM500; Ray Co., Ltd., Gyeonggi-Do, Korea). Each phantom was equipped with one of four different prostheses in the form used by a dental clinic: (1) dental amalgam restoration (Hg: 50%, Ag: 30%, Sn: 14%, Cu: 8%, 6.8 mm in height) on the right second molar; (2) gold crown (Au: 46%, Pd: 4%, Ag: 37.7%, 7.3 mm in height) on the left first molar; (3) porcelain-fused-metal (PFM) crown made of nickel-chromium ceramic alloy (Ni: 77.9%, Cr: 12.6%, Mo: 5%, Al, Be, Co, 8.1 mm in height) on the first molar; and (4) zirconia crown (ZrO_2_, 10.4 mm in height) on the left central incisor (Fig. 1).

### CBCT image acquisition

The four dental phantoms were scanned with Green21 (Vatech Co., Hwaseong, Korea), a CBCT unit equipped with MAR software. Each phantom was scanned with MAR (MAR_on_) and without MAR (MAR_off_) option under four different scan protocols; two tube voltages (70 and 100 kVp) and two voxel resolutions (0.2 and 0.3 mm^3^). The other exposure parameters were fixed as follows: field of view, 120 × 90 mm^2^; tube current, 6.5 mA; scan time, 18.0 s. The x-ray tube temperature of the CBCT scanner was kept at less than 40 °C during image acquisition to prevent image quality hindrance by tube overheating.

### Image processing and analysis

To quantify the amount of the metal-induced streak artifacts, Canny edge detection technique was modified to include noise removal as part of the whole process and applied.

**Figure 2:**
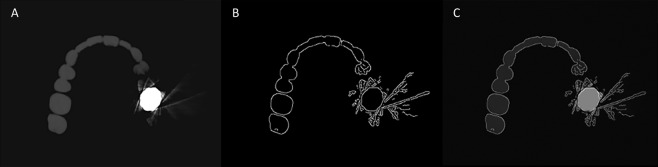
Auto-edge counting method by the application of the Canny edge detection algorithm on a CBCT image. The three images are the porcelain fused metal crown phantom obtained without the metal artifact reduction option. (**A**) Baseline image, (**B**) edge binary image using the Canny edge detection algorithm, and (**C**) superimposition of the edge binary image and baseline image. Note that the edge binary image well described the edge of the object in the baseline image.

For the image analysis, only axial slices comprising the object were included, blank upper and lower axial slices without the object were excluded. The number of axial image slices selected was as follows: (1) dental amalgam: 34 images in 0.2 mm^3^ voxel size and 22 in 0.3 mm^3^ voxel size; (2) gold crown: 46 images in 0.2 mm^3^ voxel size and 31 in 0.3 mm^3^ voxel size; (3) PFM crown: 42 images in 0.2 mm^3^ voxel size and 31 in 0.3 mm^3^ voxel size; and (4) zirconia crown: 57 images in 0.2 mm^3^ voxel size and 38 in 0.3 mm^3^ voxel size. All selected CBCT image stacks were converted into the bitmap image format without data compression or loss, yielding the baseline images (Fig. 2A). The baseline images were imported into MATLAB R2018a software (MathWorks Inc., Natick, MA, USA) and the Canny edge detection algorithm was applied to obtain edge binary images (Fig. 2B). Edge-counting was performed using MATLAB R2018a, and the result was exported into Microsoft Excel. An edge was defined as a continuous line with no breaks.

### Statistical analysis

The normality of the data was performed using the Shapiro-Wilk test when the number of selected image stacks did not exceed 30 axial slices, which resulted in a normal distribution. The edge reduction ratio was obtained for each prostheses type. The following equation was used; edge reduction ratio(%) = $\left(\frac{{\rm{Edges}}\,{\rm{in}}\,{\rm{MARoff}}\,{\rm{image}}\,-\,{\rm{Edges}}\,{\rm{in}}\,{\rm{MARon}}\,{\rm{image}}}{{\rm{Edges}}\,{\rm{in}}\,{\rm{MARoff}}\,{\rm{image}}}\right)\times 100$. Since the sample size (image slice number) was not equal, the variance homogeneity of the data was verified and Welch’s one-way analysis of variance (ANOVA) with the Games-Howell post hoc test (confidence interval = 95%) was performed to compare edge reduction ratio among each prosthesis type.

The edge count value was subtracted from MAR_off_ image by MAR_on_ image according to voxel size and tube voltage. The mean edge count of each scanning mode was compared using independent t-test (confidence interval = 95%). SPSS Statistics ver. 23.0 (IBM Co., Armonk, NY, USA) was used for all statistical analyses.

## Results

**Figure 3:**
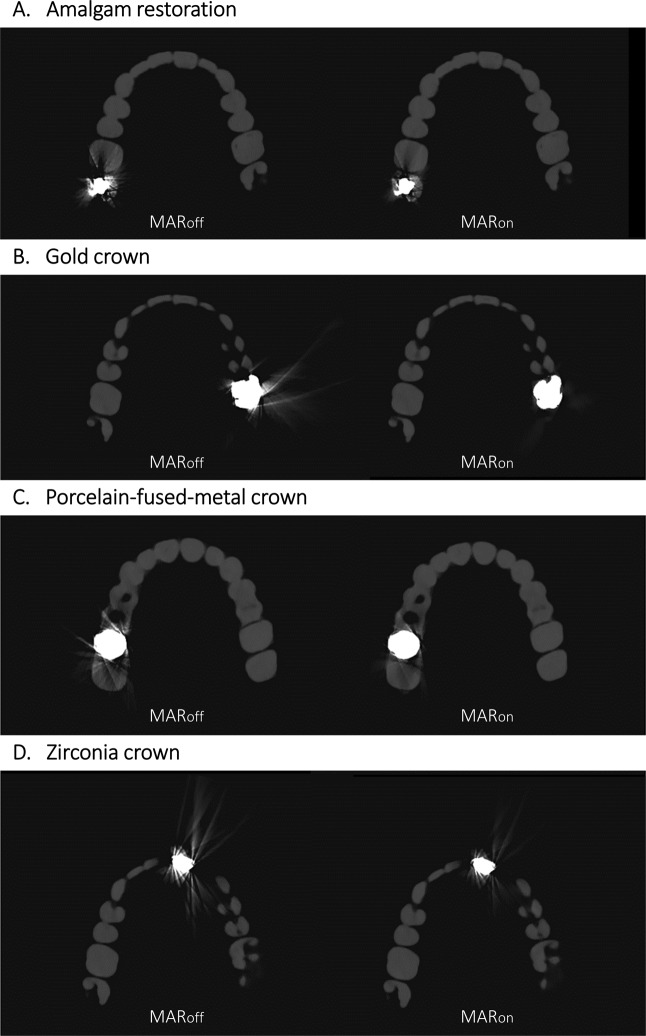
Axial images of phantoms with four different types of prostheses without and with metal artifact reduction (MAR). The scan parameters were as follows: tube voltage, 70 kVp; voxel resolution, 0.2 mm; field of view, 120 × 90 mm; tube current, 65 mA; scan time, 18.0 s.

**Figure 4:**
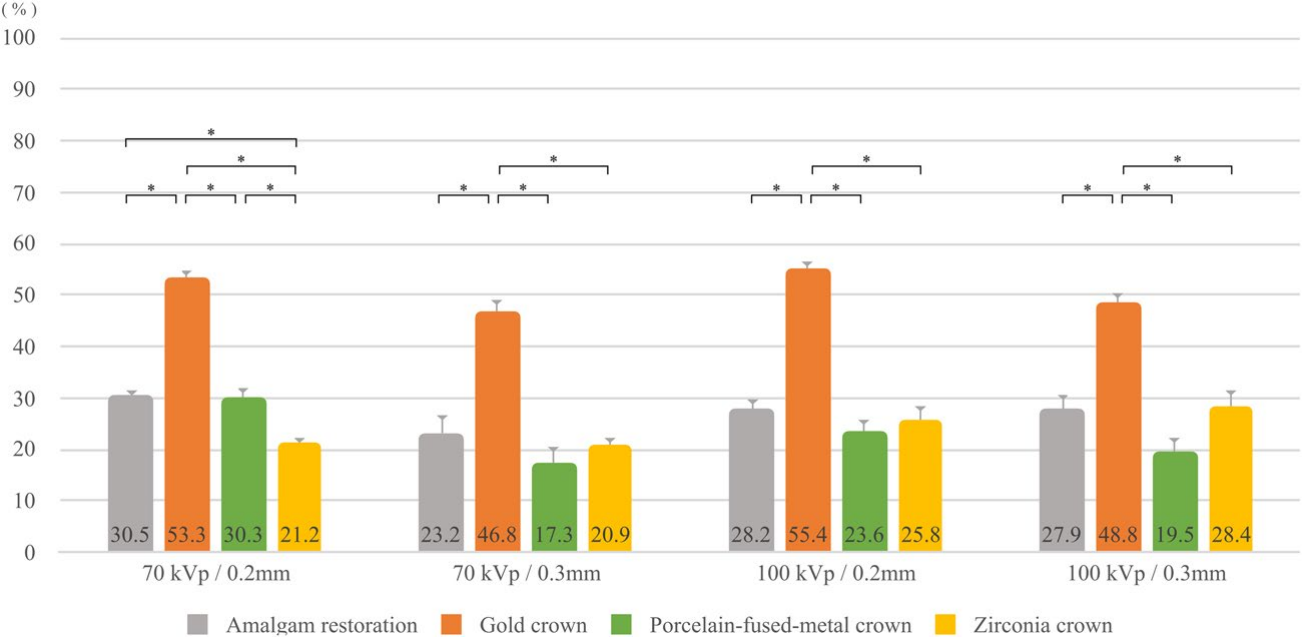
The artifacts reduction ratio (%) is calculated to compare to the prosthesis type. The ratio was calculated as the edge reduction ratio (%) = $\left(\frac{{\rm{Edges}}\,{\rm{in}}\,{\rm{MARoff}}\,{\rm{image}}\,-\,{\rm{Edges}}\,{\rm{in}}\,{\rm{MARon}}\,{\rm{image}}}{{\rm{Edges}}\,{\rm{in}}\,{\rm{MARoff}}\,{\rm{image}}}\right)\times 100$. Note that gold crown shows the highest reduction ratio with significance regardless of scanning condition. The significance in reduction ratio difference is noted with asterisk. *P-values are calculated using an one-way analysis of variance (ANOVA) with Welch’s F correction (p < 0.05).

The images obtained with MAR_on_ and MAR_off_ in the four arch-shape phantoms are shown in Fig. 3. The artifact reduction ratio was ranged from 17.3% to 55.4% according to the different prostheses of scan protocols (Fig. 4). The gold crown showed the significant reduction (*p* < 0.05) regardless of scanning mode compared to the other prostheses (Fig. 4). Among varied scanning mode, the tube voltage did not show significant effect (*p* > 0.05) on MAR while voxel size had significant correlation (*p* < 0.05) with MAR effect for all types of prosthesis. As voxel size decreased, MAR effect increased (Table 1).

**Table 1:** The number of edge difference between MAR_off_ image and MAR_on_ image according to different voxel size and tube voltage.

Prosthesis type	Voxel (mm^3^)	Tube voltage (kVp)		
		70	100	P-value
Amalgam restoration	0.2	27.18 ± 10.48	22.97 ± 10.55	0.104
	0.3	13.05 ± 10.35	14.27 ± 8.04	0.663
	P-value	0.000*	0.002*	
Gold crown	0.2	67.74 ± 33.22	66.93 ± 27.61	0.900
	0.3	35.48 ± 17.56	35.03 ± 13.59	0.910
	P-value	0.000*	0.000*	
PFM crown	0.2	34.24 ± 19.71	25.29 ± 22.56	0.056
	0.3	11.77 ± 13.53	12.45 ± 12.85	0.841
	P-value	0.000*	0.003*	
Zirconia crown	0.2	22.19 ± 7.92	22.70 ± 17.66	0.843
	0.3	14.26 ± 6.57	16.82 ± 9.97	0.192
	P-value	0.000*	0.041*	

MAR; metal artifact reduction, PFM; porcelain-fused-metal.

Data are presented as mean ± standard deviation.

*p-value by independent t-test.

## Discussion

Metal artifacts on images, which appear as white and dark bands, can significantly degrade the quality of CBCT images^4,5,7,8^. Cupping artifact, also induced by the dense metal, occurs as the x-ray over-attenuated on the center of the dense object compared to the periphery. This artifact causes inaccuracy of the object size^9^. Basically, MAR algorithm was developed to reduce those artifacts induced by metals. The algorithm eliminates voxels with too high or too low grey values and reconstruct this area until they have similar values as nearby voxels^8^. Streak artifacts which appear more extensive in image would be more effectively eliminated compared to the cupping artifacts. Still, it might also have positive influence on cupping artifact correction, although it is hard to quantify separately from the streak artifact.

Since the metal artifacts appear more intensely when dense material has a higher atomic number and a complex shape, they are an issue to be solved in areas of dentistry where various types of prostheses are used for treatment^5,12^. Different prosthetic materials induce different amounts of artifacts^5,23^. Most previous researchers studied metal artifacts using various type of metal rods inserted into phantoms^16,24^. These studies mentioned the necessity of a study that considers the frequently used materials and their shape and locations in dental arch reflect the clinical situation. Thus, the present study focused on the metal artifacts in various shape, size and location of dental material. The dental materials are diverse in clinical usage according to the tooth type and restoration extent. For example, gold alloy is mainly used in the posterior tooth as partial restorative material or crown. Amalgam is also used mainly in the posterior tooth while it is not used as crown. In the present study, we fabricated phantoms with an arch-shape base and four prostheses inserted along the dentition according to their main purposes in dental arch to reflect the clinical setting. Therefore, despite the controlled setting of present experiment, the limitation should be understood of using the differences in size and shape of each prosthesis.

Additionally, we studied zirconia, which is a non-metal that nonetheless has a high atomic number and is being used with ever-increasing frequency. The zirconia crown induced an amount of artifacts comparable to those induced by the metal materials, and the MAR algorithm was effective without exception. The amount of artifact reduction differed by the material type of the prosthesis. The artifacts from the gold crown were more efficiently removed than those from the other materials. This might be due to the fact that the gold alloy is the most frequently used material in dental field and MAR algorithm of CBCT was developed based on this clinical situation. The other materials (amalgam restoration, PFM crown, and zirconia crown) showed lower levels of reduction compared to the gold crown, while the reduction ratio was varied according to the tube voltage and voxel resolution.

The high tube voltage is known to reduce metal artifact in CT^11,17^. This is because high tube voltage induces less beam hardening tendency. In this study, there were no tendency in MAR efficiency according to the tube voltage. This was probably due to that the amount of tube voltage increased was not enough to change beam hardening tendency which is caused by dental prosthesis with exceedingly high density.

Voxel size (determined by matrix, FOV, and slice thickness) is known to be closely related to metal artifact reduction on CT images^13,25^. In CBCT, voxel size effect on MAR was controversial. Recent study on CBCT metal artifact reported that voxel size did not show significant effect on metal artifact reduction for amalgam and copper-aluminum alloy^19^. In the current study, as voxel size decreased MAR algorithm efficiently eliminated artifact more regardless of prosthesis type. The disagreement of these results might be due to the difference in artifact quantification methods.

An objective and quantitative evaluation of the efficacy of the MAR in CBCT is challenging. Previous studies were performed based on gray-level measurements in the region of interest (ROI) to quantify the amount of artifacts^16–19^. However, CBCT images do not always show constant gray values for a single material, even with the same equipment^26,27^. Moreover, gray values in CBCT images depend on various factors, including exposure conditions, the patient’s position, the machine model, and even room temperature, but are especially sensitive to the area or size of the ROI^21,28^. In the present study, quantification of metal artifact was performed based on the method less dependent on gray value of image. The edge detection method was utilized for quantification of the white streak band in CBCT images for this study. The method succeeded in presenting the MAR effect in numerical form, which was consistent with the results of a visual inspection. This technique is also expected to be useful for comparing MAR efficacy between different CBCT machines.

Although the varied size and shape of the metal materials of the present study affect the artifact evaluation results, the findings from this study may be helpful to clinicians since the fabricated phantom reflects the actual clinical conditions. However, the artifact due to patient skull anatomy and soft tissue was not considered in here, therefore, for the further study, this should be supplemented. Then, the quantitative method presented in this paper will help to evaluate and compare different MAR algorithms for various CBCT systems and settings in order to identify the most effective algorithm.

## Conclusion

This study found that the MAR algorithm of the CBCT device efficiently reduced streak artifacts substantially, albeit to a different degree for individual prostheses, and it was most efficient for the gold crown. The impact of voxel size on metal artifact reduction was significant and that of tube voltage was unclear, suggesting that thoughtful consideration is required when adjusting these conditions for CBCT examinations of patients with various dental prostheses. The auto-edge counting method can be useful for quantitative evaluation of MAR in CBCT images.
